# P-2091. Nirsevimab uptake and RSV-associated outcomes among infants in the US

**DOI:** 10.1093/ofid/ofaf695.2255

**Published:** 2026-01-11

**Authors:** Emma Guare, Paddy Ssentongo, Cory M Hale, Jessica E Ericson, Catharine I Paules

**Affiliations:** Penn State College of Medicine, Hershey, Pennsylvania; Penn State Health Milton S. Hershey Medical Center, Hershey, PA; Penn State Health Milton S. Hershey Medical Center, Hershey, PA; Penn State College of Medicine, Hershey, Pennsylvania; Penn State Health Milton S. Hershey Medical Center, Hershey, PA

## Abstract

**Background:**

The monoclonal antibody nirsevimab was approved in July 2023 for prevention of respiratory syncytial virus (RSV) in infants, a public health priority. To date, only one single-institution study has reported on nirsevimab uptake, reporting 70.1% among eligible infants (Blauvelt 2025). There is an overall lack of understanding about nirsevimab uptake throughout the US regarding differences in access, demographics, and region. Real-world effectiveness of RSV prevention modalities is also limited.

**Table 1.**
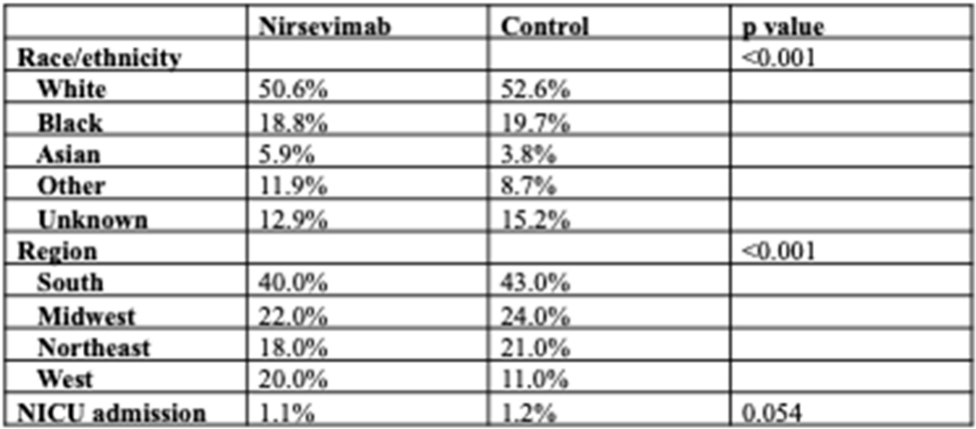
Infant characteristics between those who received nirsevimab and propensity-matched controls

**Figure 1.**
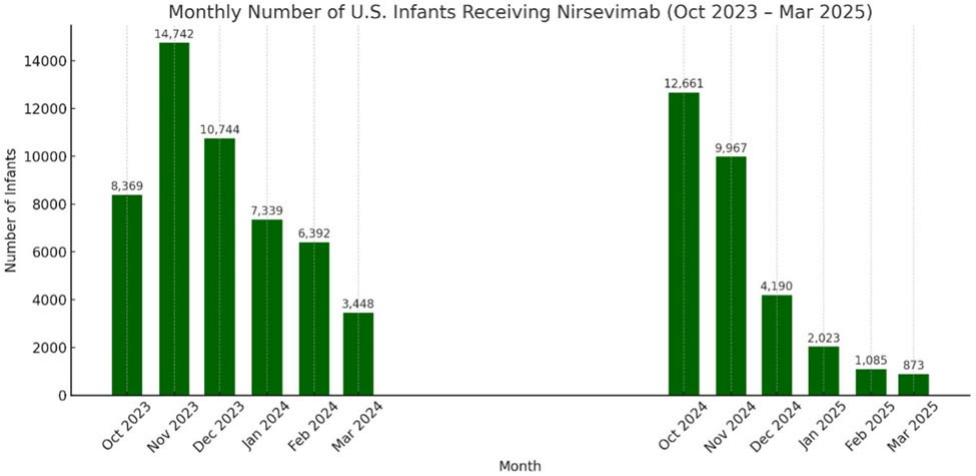
Nirsevimab uptake by in the United States via TriNetX The bar plot shows the monthly uptake of nirsevimab across two RSV seasons.

**Methods:**

This is a retrospective cohort study using TriNetX US Collaborative Network data of infants under 1 year who received nirsevimab between October 2023 and March 2025. We matched infants who received nirsevimab 1:1 with infants who did not based on age at nirsevimab receipt. Infant characteristics, month of nirsevimab administration, and RSV-related outcomes were analyzed.

**Figure 2.**
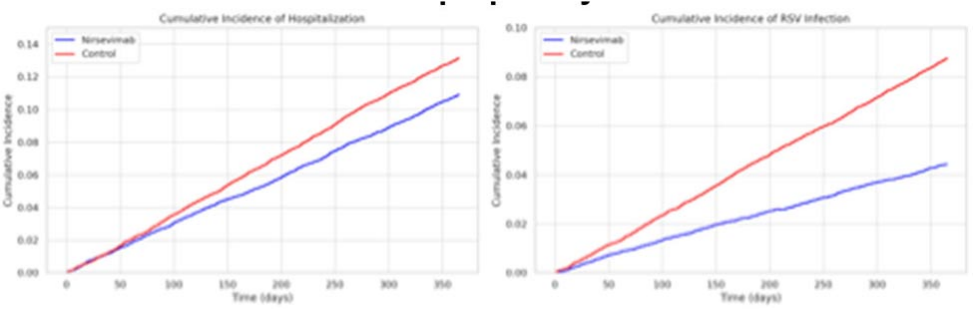
Cumulative incidence of any hospitalization and RSV infection between infants who received nirsevimab and propensity-matched controls. The y-axis represents cumulative incidence, and the x-axis indicates days from nirsevimab dose and age at time of matching for the control infant.

**Figure 3.**
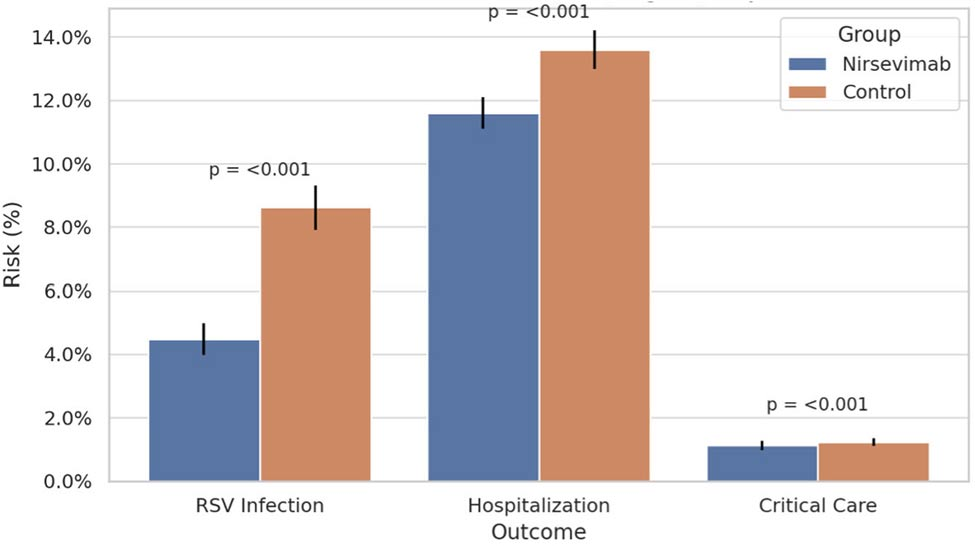
Risk of RSV infection, any hospitalization, and critical care admission between infants who received nirsevimab and propensity-matched controls. The y-axis represents the percentage risk of the outcomes.

**Results:**

A total of 38,958 infants under 1 year received nirsevimab. Compared to controls, more recipients were Asian (5.9%) and from the West (20%, Table 1). Nirsevimab administration peaked in November 2023 and October 2024 across the past two RSV seasons (Fig 1). Recipients had significantly lower rates of RSV infection and any hospitalization over time (Fig 2), along with fewer critical care admissions compared to controls (Fig 3).

**Conclusion:**

Nirsevimab was associated with improved RSV-related outcomes compared to controls in our dataset and in published efficacy and effectiveness studies (Moline 2025, Lefferts 2025). Thus, it is critical to understand barriers to administration in the real world. In our dataset, there was variation in nirsevimab uptake by race/ethnicity and region, highlighting the need for more granular analysis. Interestingly, a higher proportion of infants received nirsevimab in the West compared to controls. Given that the only real-world study thus far has been conducted in the West and showed high uptake^1^, barriers there may be less than in other regions. There are important limitations to our data due to the nature of the TriNetX dataset, including the inability to account for maternal RSVpreF vaccination. In future work, we aim to link infant-mother pairs and assess in greater detail the social determinants that affect uptake of both FDA-approved RSV prevention modalities.

**Disclosures:**

All Authors: No reported disclosures

